# Prolonged Methylprednisolone Therapy in the Fibro-Proliferative Phase of Acute Respiratory Distress Syndrome

**DOI:** 10.7759/cureus.19906

**Published:** 2021-11-25

**Authors:** Derya Kocakaya, Sehnaz Olgun Yıldızeli, Çiğdem Ataizi-Çelikel, Berrin Ceyhan

**Affiliations:** 1 Pulmonary and Critical Care Medicine, Marmara University School of Medicine, Istanbul, TUR; 2 Pathology, Marmara University School of Medicine, Istanbul, TUR

**Keywords:** prolonged methylprednisolone therapy, acute respiratory distress syndrome, respiratory distress syndrome, methylprednisolone, pulmonary fibrosis

## Abstract

Late-stage acute respiratory distress syndrome (ARDS), primarily associated with fibro-proliferative changes, may occur in many patients. This stage, where ARDS progresses to the point of being incurable, involves a complicated and long clinical course that may give rise to functional loss; it has therefore been a major focus of both preventive and therapeutic strategies. In the present case report, the successful use of prolonged methylprednisolone therapy in the fibro-proliferative phase of ARDS is described in a patient who developed pneumonia and secondary ARDS after terminating a pregnancy due to preeclampsia. Methylprednisolone therapy, which was initiated at a daily dosage of 1 mg/kg, was tapered down based on the clinical and radiologic status of the patient and was terminated at the end of the sixth month. Follow-up imaging studies and pulmonary function tests performed at the end of the first and sixth months showed marked improvements and the patient experienced no systemic adverse effects despite long-term steroid therapy.

## Introduction

Acute respiratory distress syndrome (ARDS) is a condition characterized by acute hypoxemic respiratory failure secondary to inflammatory lung injury [1].

ARDS may follow different clinical courses; some patients recover quickly, but others may develop subsequent pulmonary inflammation and fibrosis [2-3]. Late-stage ARDS, primarily associated with fibro-proliferative changes, may occur in many patients and may be accompanied by ongoing hypoxemia, fever, leukocytosis, and pulmonary inflammation without infection, and result in an increased risk for fibrosis [2]. This stage, where ARDS progresses to the point of being incurable, involves a complicated and long clinical course that may give rise to functional loss; it has therefore been a major focus of both preventive and therapeutic strategies. Several anti-inflammatory and immunomodulatory agents have been investigated for the treatment of ongoing inflammation and fibrosis, among which, corticosteroids appear to be the most emphasized and discussed [2,4-6].

In the present case report, the successful use of prolonged methylprednisolone therapy in the fibro-proliferative phase of ARDS is described in a patient who developed pneumonia and secondary ARDS after terminating a pregnancy due to preeclampsia.

## Case presentation

A 28-year-old female patient presented to a physician at the 33rd gestational week with symptoms of hypertension and accompanying edema. Laboratory tests showed nephrotic range proteinuria and the patient was hospitalized after being diagnosed as having preeclampsia. The pregnancy was terminated by cesarean section. She developed a fever and respiratory distress on postoperative day two. Antibiotic treatment was initiated with a diagnosis of pneumonia. The patient experienced increased respiratory distress during the follow-up period and was transferred to the intensive care unit with a suspected diagnosis of acute respiratory failure. The patient was connected to non-invasive mechanical ventilation (NIMV). A thorax CT scan showed symmetrical consolidated regions in the basal and perihilar segments of both lungs, with air bronchograms pronounced in the dependent regions, in addition to minimal pleural effusion in both lungs and a global increase in heart size. The upper lobes of both lungs and the middle lobe of the right lung were conserved, and the findings were consistent with pneumonia on the background of pulmonary edema. The patient was given bronchodilators and diuretic therapy, but a follow-up thorax CT showed an increase in ground-glass opacities so the patient was intubated due to worsening respiratory distress. Laboratory investigations performed at that stage indicated hypoalbuminemia and anemia.

Her C-reactive protein (CRP) levels decreased under antibiotic treatment and respiratory parameters improved shortly after initiation of mechanical ventilation. She was therefore extubated and supported by NIMV. But her proteinuria was 18 g/day and she was transferred to another center to be followed up by the nephrology department. There, the patient once more experienced respiratory distress and developed peripheral cyanosis. She was re-intubated and monitored in the intensive care unit. A repeat thorax CT showed pneumothorax, which was minimal on the left side (less than 5%), and pneumomediastinum. The patient was extubated after the improvement of respiratory parameters and monitored under oxygen support provided using a nasal cannula. The pneumonia was not considered to be improving based on her ongoing need for oxygen and increases in leukocytosis and CRP levels. Consequently, the antibiotic regimen was changed and the patient, who had ongoing proteinuria and hypoalbuminemia during the entire follow-up period of almost one month, was transferred to our hospital to be monitored by the nephrology department.

At admission, the patient’s general condition was good and she was in need of 4 L/min oxygen support. Her chest radiograph showed bilateral extensive reticulonodular infiltrates and a previously obtained thorax CT scan was found consistent with hypervolemia accompanied by infective pathology. Subsequently, as the patient’s CRP levels decreased and fever resolved, the antibiotics were stopped after completing a ten-day course.

**Figure 1:**
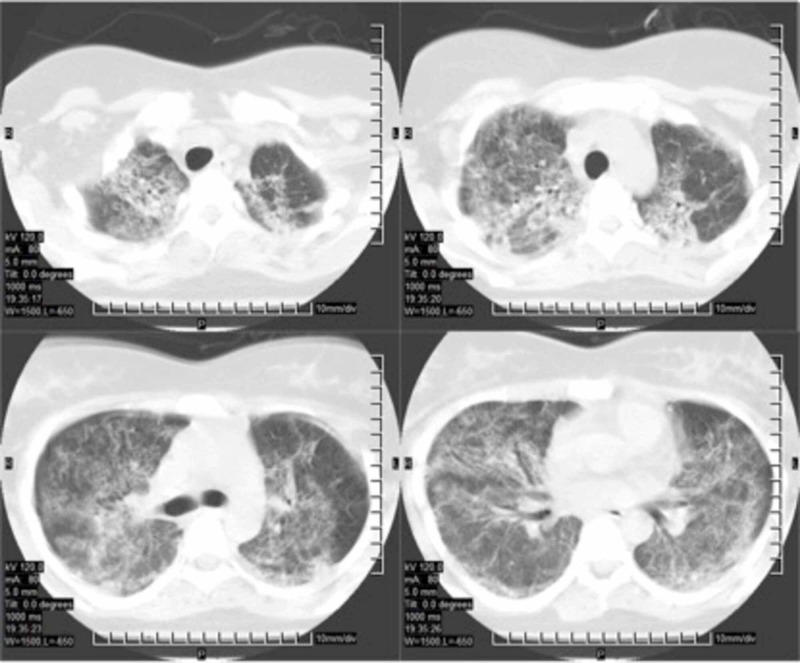
Thorax CT images of the patient before corticosteroid treatment showing bilateral extensive ground-glass opacities with adjacent traction bronchiectasis and interlobular septal thickenings.

The patient’s protein level in a 24-hour urine sample was 3.5 g/day and urine sediment showed an average of 5-6 isomorphic erythrocytes, rare leukocytes, and an abundance of hyaline cylinders per field. Based on the recommendations of the Department of Nephrology, 1500 cc fluid restriction was started. The patient was given furosemide infusion because her physical examination indicated bilateral rales. At this time, cardiac evaluation was normal, and resolving renal condition was attributed to pregnancy, and no further evaluation was suggested. Her oxygen requirement decreased but did not resolve, thus a repeat thorax CT scan was obtained, which showed extensive bilateral traction bronchiectasis, adjacent to the regions of extensive ground-glass opacities, and thickening of the interlobular septa (Figure 1).

**Figure 2:**
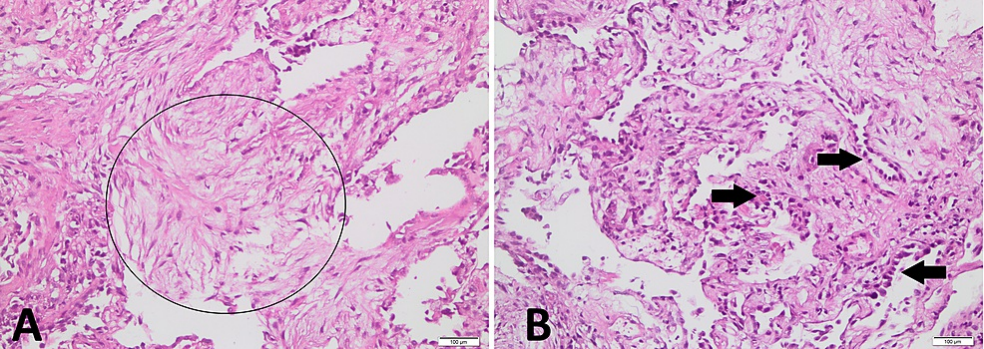
Hematoxylin and eosin staining, 20X: (A) Fibroblastic proliferation marked with circle; (B) Type II pneumocyte hyperplasia and hypertrophy marked with arrows

These findings were considered to be consistent with interstitial pneumonia and 60mg/day methylprednisolone therapy was initiated. The patient’s need for oxygen ended on the second day of steroid therapy. Rheumatologic pathologies, cryptogenic organized pneumonia, and fibro-proliferative phases of ARDS were considered as differential diagnoses. Rheumatologic evaluations revealed no pathologies, and she was negative for ANA and anti-ds-DNA. Thus, bronchoscopy was performed with the aim of establishing a tissue diagnosis on the second day of steroid treatment. No endobronchial lesion was detected, bronchoalveolar lavage fluid revealed no microbiological pathogens and pathologic examination of the transbronchial biopsy obtained from the right lower lobe showed fibroblastic proliferation, type II pneumocyte hypertrophy, and hyperplasia of the alveolar septa (Figure 2), which were consistent with the resolution period of diffuse alveolar damage.

**Figure 3:**
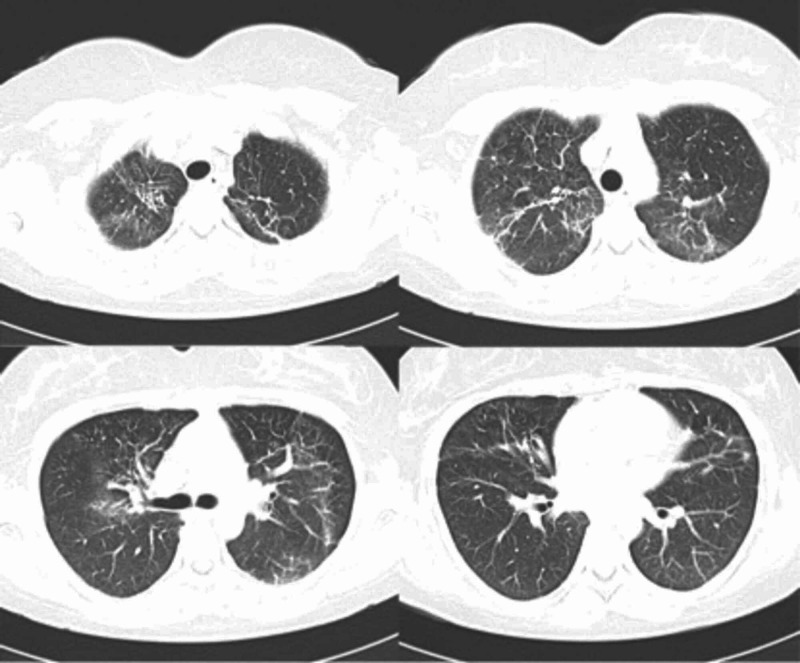
Thorax CT images of the patient after corticosteroid treatment showing resolution of nearly all ground-glass opacities.

A thorax CT obtained at the end of the first month of steroid therapy showed regression in the previously observed regions of ground-glass opacity. However, as the patient described exertional dyspnea and her pulmonary function tests showed decreased forced vital capacity (FVC), it was planned to continue steroid therapy at a lower dose. The methylprednisolone dose was reduced to 24 mg at the end of 1 month by reducing the dose once a week and stopped after a period of six months, during which the patient experienced no adverse effects except for plethoric skin. The patient’s symptoms were completely resolved, her FVC increased from 55% to 78%, and almost all ground-glass opacities on thorax CT disappeared (Figure 3); she is currently being followed up without any medical therapy.

## Discussion

ARDS occurs in response to a rapidly developing diffuse damage in the alveoli and it is one of the most common causes of hypoxemic respiratory failure [7]. The resulting diffuse alveolar damage triggers the release of several pro-inflammatory cytokines. These cytokines induce migration of neutrophils to the lungs, where they are activated and cause endothelial damage in the capillaries and alveoli through the effects of toxic mediators [8]. This extensive microcirculation damage associated with ARDS results in leakage of protein-rich fluid into the pulmonary interstitium and alveolar space. These changes appear as bilateral extensive infiltrates on chest radiographs, and lead to severe hypoxemia as well as a decrease in thoracic compliance [2].

Patients with ARDS go through three relatively different pathologic phases. The initial stage, called the exudative phase, is characterized by diffuse alveolar damage. The proliferative phase, which starts within seven to 10 days, involves a fibro-proliferation reaction characterized by myofibroblast aggravation and collagen accumulation in the lungs and results in the transformation of the protein-rich exudate produced during the first phase into granulation tissue. Progressive fibro-proliferation is a direct cause of death in 15-40% of the patients, but it may also result in death indirectly, by prolonging the patients’ need for ventilatory support and thus predisposing them to nosocomial infections, primarily pneumonia [4]. The outcomes of this phase, referred to as late-stage ARDS, can be improved by reducing the risk for potential complications by preventing the progression of fibro-proliferation, suppressing fibrosis, and shortening the duration of ventilatory support [2]. In patients with ARDS who have extensive fibrosis as demonstrated in an open lung biopsy, the fibro-proliferative phase can be partially reversed despite a decreased response to mechanical ventilation and reduced survival [9].

Corticosteroids may prove useful in this respect, by regulating the functions of macrophages and fibroblasts [2]. In addition, they may improve gas exchange by reducing the levels of pro-inflammatory mediators in the lungs and circulating in serum, thereby decreasing lung injury as well as the need for mechanical ventilation [10-13].

Ashbaugh et al. were the first to demonstrate the development of apparent cellular proliferation, alveolar deletion, and fibrosis based on open lung biopsy, on an average of eight days after onset of ARDS in a group of 10 patients with postoperative ARDS accompanied by fever, leukocytosis, and abnormal respiratory parameters in the absence of an infection. Corticosteroid therapy provided clinical and physiologic improvement in these patients and resulted in a survival rate of 80% [14]. Later, Hooper et al. administered corticosteroid therapy in the absence of a histologic diagnosis in 10 patients who survived early-phase ARDS but had extensive bilateral lung involvement as demonstrated by gallium scintigraphy without any findings of infection and reported clinical as well as radiologic improvement during follow-up [15]. Both groups of researchers underlined that early termination of steroid therapy resulted in rapid deterioration of gas exchange, and an improvement could be achieved upon re-initiation of therapy [14-15].

In a study by Meduri et al. in 1991, the fibro-proliferative phase of diffuse alveolar damage was demonstrated in open lung biopsies in seven out of eight patients with ARDS. High-dose corticosteroid therapy, used for a mean duration of 36 weeks in this patient group, provided improvements in clinical findings in addition to inflammatory parameters such as fever, neutrophil count in bronchoalveolar lavage, and the extent of involvement as demonstrated by gallium scintigraphy [2]. In another study published in 1994, the same researchers initiated methylprednisolone therapy in 25 patients at a daily dosage of 2-3 mg/kg on the seventh to 21st day of ARDS, and then they gradually reduced the dose until cessation within six weeks. The authors reported significant improvements in the patients’ clinical course after steroid therapy, accompanied by a reduced need for oxygen support and mechanical ventilation, improved chest radiography findings, and decreased inflammation [4]. The researchers also found steroid therapy more effective in patients whose open lung biopsies did not establish acellular fibrosis. In another study, which included 24 patients, the effects of adding corticosteroids to the treatment regimens of non-responsive ARDS patients on the seventh day of ARDS were investigated, and patients receiving steroids showed better lung functions, decreased mortality, and less multi-organ dysfunction compared with those who did not use steroids [10]. On the other hand, these data were not supported by a study published in 2006, which failed to show a decrease in the mortality rate or the number of days without ventilatory support upon the addition of methylprednisolone to treatment regimens of 180 patients after the seventh day of ARDS [6].

Conflicting results have been reported on the role of steroids in the course of ARDS, and meta-analyses of all previous studies were performed to clarify these conflicts. These meta-analyses concluded that despite positive results obtained with the use of steroids in ARDS, these data should be further supported by large-scale, randomized, controlled trials, and the role of steroids in ARDS treatment is still controversial based on available data [5,16-19].

The present case report described a patient who developed ARDS secondary to pneumonia after the termination of pregnancy due to preeclampsia. The patient had an ongoing need for mechanical ventilation for almost two weeks, followed by one month of the continuous need for oxygen therapy. Different interstitial diseases and especially the fibro-proliferative phase of ARDS were considered as differential diagnoses because the patient’s thorax CT scan suggested that her condition was progressing to fibrosis. Pathologic examination of the transbronchial biopsy samples indicated the resolution phase of diffuse alveolar damage.

Methylprednisolone therapy, which was initiated at a daily dosage of 1 mg/kg, was tapered down based on the clinical and radiologic status of the patient and was terminated at the end of the sixth month. Follow-up imaging studies and pulmonary function tests performed at the end of the first and sixth months showed marked improvements and the patient experienced no systemic adverse effects despite long-term steroid therapy.

## Conclusions

ARDS, which has serious and life-threatening consequences, especially in the acute process, is of serious importance in terms of causing permanent damage to the lung in the late period. Although treatment strategies are still controversial; current literature about systemic steroids is promising for late-stage ARDS. However, large-scale, randomized, controlled trials are still required to support this finding.
